# Bacterial contamination on mobile phones in the theatre area of a large veterinary hospital

**DOI:** 10.1002/vro2.70016

**Published:** 2025-08-19

**Authors:** Owen J. Glenn, Lisa Telford, Kathryn M. Pratschke

**Affiliations:** ^1^ Hospital for Small Animals The Royal (Dick) School of Veterinary Studies and The Roslin Institute The University of Edinburgh Edinburgh UK

## Abstract

**Background:**

Mobile phones are commonly used in clinical areas, including theatre, anaesthesia and patient preparation. Contamination rates of 72.6%–100% for mobile phones are reported in human healthcare, including multidrug‐resistant bacteria in more than 30%. Veterinary studies are limited to *Staphylococcus* only, but similarly high levels of contamination are reported.

**Methods:**

The personal and work mobile phones of veterinarians and nurses were swabbed for bacterial culture. Gram morphology and colony‐forming units per millilitre (CFU/mL) were recorded for each bacteria grown. Participants completed an anonymous survey detailing habits of phone usage and hygiene.

**Results:**

Swabs from 39 mobile phones were collected, with 16 of 39 (41%) positive for bacterial contamination. Gram‐positive cocci were most common (38.4%), with 5.13% Gram‐positive coccobacilli, 5.13% Gram‐positive rods and 2.56% Gram‐negative rods. Veterinarians’ mobile phones had a significantly higher incidence of contamination than nurses’ (62.5% vs. 6.7%; *p* = 0.007). More personal phones were contaminated than work phones (52.6% vs. 30%). Two mobile phones had growth of colonies more than 4000 CFU/mL. Mobile phone usage in clinical areas was reported by 76.2% of 21 participants, 57.1% cleaned their mobile phone less than weekly, 23.8% used non‐antibacterial items to clean with and 23.8% had recently used their mobile phone in the bathroom.

**Conclusions:**

Bacterial contamination of mobile phones was common, with veterinarians having a significantly higher incidence compared to nurses. Gram‐positive cocci were most common, but Gram‐positive coccobacilli, Gram‐positive rods and Gram‐negative rods were also present. Mobile phone hygiene could be usefully included in infection control guidelines as they are potential fomites for healthcare‐associated infections.

## INTRODUCTION

Mobile phones are almost ubiquitously used by healthcare workers for patient‐side tasks.^1^, ^2^, ^3^, ^4^, ^5^ They can have a positive impact on patient care,^2^ but concerns have been raised over their potential to act as a fomite for transmission of infection and provide a new challenge in infection control.^3^, ^4^, ^5^, ^6^, ^7^, ^8^, ^9^, ^10^, ^11^, ^12^, ^13^, ^14^, ^15^, ^16^, ^17^, ^18^, ^19^, ^20^, ^21^, ^22^, ^23^

Bacterial contamination of mobile phones has been extensively documented in human healthcare, with reported contamination rates of 72.6%–100% in operating theatres and critical care units.^3^, ^6^, ^7^, ^11^, ^17^, ^18^, ^20^, ^22^, ^23^, ^24^ Bacterial species regularly identified include *Staphylococcus* spp., *Klebsiella* spp., *Clostridium difficile*, *Enterococcus faecium*, *Pseudomonas aeruginosa*, *Acinetobacter* spp., *Bacillus* spp. and *Neisseria* spp.^6^, ^11^, ^17^, ^18^, ^20^, ^21^, ^22^, ^23^ Bacteria with antibiotic‐resistant characteristics have been reported from 31% to 100% of phones sampled in various studies.^6^, ^11^, ^17^, ^18^, ^20^, ^21^, ^22^, ^23^ Two studies have evaluated mobile phone and tablet contamination in veterinary hospitals; however, both assessed only *Staphylococcus* spp.^4^, ^12^ Staphylococcal contamination was found in 68%^4^ and methicillin‐resistant *Staphylococcus aureus* (MRSA) or *Staphylococcus pseudintermedius* (MRSP) were found in 2.4%.^12^ No studies to date have evaluated the non‐staphylococcal contamination of mobile phones in veterinary hospitals.

Fomites are an important reservoir of bacteria that are linked to healthcare‐associated infections (HAIs)^25^ and increased hand hygiene reduces these infections.^26^, ^27^ While no direct causation has been established between mobile phone contamination and HAIs,^4^, ^10^ mobile phone colonisation closely resembles hand contamination,^21^ which is known to directly affect HAIs.^26^, ^27^ Other innocuous items such as keyboards, toys and even soap dispensers have been identified as causes of multidrug‐resistant (MDR) HAI outbreaks in human healthcare.^28^, ^29^, ^30^ Therefore, mobile phones may act as a reservoir for bacterial transmission to patients, causing HAIs. Surgical site infections are one of the most common and significant HAIs.^31^

The primary aim of this study was to evaluate microbial contamination, beyond Staphylococcal species, on mobile phones used by veterinarians and nurses within the theatre, patient preparation and anaesthesia areas of a large veterinary teaching hospital. The secondary aim was to investigate the habits of mobile phone use and cleaning.

## MATERIALS AND METHODS

### Enrolment and data collection

Participants were prospectively enrolled in this cross‐sectional study at a single time point. Inclusion criteria were veterinarians and nurses present at the time of sample collection in the theatre, patient preparation and anaesthesia areas of a single veterinary teaching hospital. Exclusion criteria were students, visitors and people without a mobile phone available for sampling. No advanced warning was given and sampling was undertaken on one occasion to ensure the study reflected normal behaviour and not a Hawthorne effect, where the knowledge of prospective sampling may have caused participants to change their mobile phone hygiene habits.^32^ All participants were informed of the study and consented to participate, with sampling undertaken immediately after signed consent was obtained. Enrolment and sample collection were conducted between 10:30 AM and 12:00 PM on 20 March 2024. All eligible people were invited to participate until 40 samples were collected, based on sampling of two mobile phones from 80% of the estimated average staff numbers present and the available funding.

Participants were categorised by hospital department and veterinarian or nurse. Mobile phones were categorised into ‘personal’ or ‘work’. ‘Work’ phones were all hospital‐provided non‐touchscreen phones without internet connectivity (DECT G277, NEC).

Bacterial samples were collected using new non‐sterile nitrile gloves and a plain sterile cotton swab (Scientific Swab Plastic PlainTube, Thermo) for each mobile phone. Swabs were moistened with three to five drops of sterile saline and wiped and rotated to expose the whole swab across all surfaces, ensuring the screen, case, microphone, buttons and speakers were sampled. Swabs were then placed into their accompanying sterile transport tubes and transported directly to the on‐site microbiology laboratory for processing within 90 min of collection. Participants were then asked to complete an anonymous online survey to help characterise general habits around mobile phone usage (Appendix 1). Survey responses could not be directly linked to individual bacterial swab results due to limitations set by ethical approval.

### Microbiological data

At the microbiological laboratory, each swab was cut to length with sterile scissors, placed in a bijou bottle containing 3 mL of sterile saline (0.85% Sterile Saline, E&O Laboratories) and vortexed for 10 s. Fifty microlitres of the resulting solution was added to the centre of a Columbia Horse Blood Agar Plate (E&O Laboratories) and spread over the entire surface of the plate. Plates were incubated aerobically at 37°C overnight. The following day each plate was checked for growth, colony types were noted and colony‐forming units (CFUs) were counted. A maximum of 200 CFUs per colony were manually counted and quantities above this were recorded as more than 200. CFU was multiplied by the dilution factor of 20 to achieve CFU/mL. Colony types were checked microscopically for their Gram morphology (e.g., Gram‐positive cocci). Plates were re‐incubated overnight; any additional growth was noted the following day and any ‘new’ colony types were processed as above.

### Statistical analysis

Data were collated in Excel (Excel 16.56, Microsoft) and analysed in GraphPad Prism (Prism v9.5.1 for macOS, GraphPad Software). Continuous data were assessed for normality using Shapiro‒Wilk tests and found that no data were normally distributed. Continuous data are therefore presented as median with range and categorical data as frequency with range. Continuous data with two groups were analysed using Mann‒Whitney *U*‐tests, and continuous data with three or more groups were analysed using Kruskal‒Wallis tests. Categorical data with two groups were analysed using Fisher's exact tests and categorical data with three or more groups were analysed using chi‐squared tests. Statistical significance was set at *p*‐value of less than 0.05.

## RESULTS

### Bacterial swabs

Thirty‐nine mobile phones from 25 participants were included. One swab was excluded due to accidental contamination during sampling. The number of participants, mobile phones, positive bacterial swabs and total CFU/mL per positive swab by department, role and type of mobile phone are presented in Table 1. The highest contamination rate came from the subcategory ‘surgery—veterinarian’ with 10 of 13 (76.9%) positive swabs. This was followed by ‘anaesthesia—veterinarian’ at five of nine (55.6%) positive, and then ‘anaesthesia—nurse’ at one of five (20%). ‘Surgery—nurse’, ‘emergency and critical care—veterinarian’ and ‘emergency and critical care—nurse’ had no positive swabs. There was a significant difference between the frequency of positive swabs between mobile phones from veterinarians and nurses (*p* = 0.0007; Table 1), with veterinarians being significantly more likely to have a contaminated mobile phone. There was no significant difference between the frequency of positive swabs or total CFU/mL per positive swab by department (*p* = 0.32 and 0.95) or mobile phone type (*p* = 0.2 and 0.52). Due to the limited numbers in each subgroup, further detailed analysis between subgroups was not performed.

**TABLE 1:** Number of participants, mobile phones and positive bacterial swabs by participant department, role and mobile phone type.

Category	No. of participants	No. of mobile phones	No. of positive swabs	Percent of positive swabs	Total CFU/mL per positive swab, median (range)
Department					
Surgery	15	22	10	45.5%	60 (20 to >8460)
Anaesthesia	8	14	6	42.9%	50 (20 to >8000)
ECC	2	3	0	0%	0
Role					
Veterinarians	15	24	15	62.5%^a^	60 (20 to >8460)
Nurses	10	15	1	6.7%^a^	40
Combined department and role					
Surgery—veterinarian	9	13	10	76.9%	60 (20 to >8460)
Surgery—nurse	6	9	0	0%	0
Anaesthesia—veterinarian	5	9	5	55.6%	60 (20 to >8000)
Anaesthesia—nurse	3	5	1	20%	40
ECC—veterinarian	1	2	0	0%	0
ECC—nurse	1	1	0	0%	0
Mobile phone type^b^					
Personal	19	19	10	52.6%	50 (20 to >8460)
Work	20	20	6	30%	40 (20 to >8000)
Total	25	39	16	41%	60 (20 to >8460)

Abbreviations: CFU, colony‐forming units; ECC, emergency and critical care.

^a^ Significant difference between groups, *p* = 0.0007.

^b^ Participants could contribute both a personal and work mobile phone.

The Gram morphology, number of positive bacterial swabs and CFU/mL per colony are presented in Table 2. Multiple bacterial isolates were cultured from six mobile phones; four had two isolates, one had three isolates and one had five isolates. Seven mobile phones had a CFU/mL per colony above twice the median, with two mobile phones having more than 4000 CFU/mL per colony. Both had two bacteria at this level; three were Gram‐positive cocci and one was a Gram‐negative rod.

**TABLE 2:** Gram morphology and colony‐forming unit (CFU)/mL for 16 positive bacterial swabs taken from 39 mobile phones.

Gram stain	Morphology	No. of isolates	No. of positive swabs^a^	Percent of positive swabs	CFU/mL per colony, median (range)
Positive	Cocci	21	15	38.4%	60 (20 to >4000)
Positive	Coccobacilli^b^	2	2	5.13%	30 (20‒40)
Positive	Rods^b^	2	2	5.13%	40 (40‒40)
Negative	Rods^b^	1	1	2.56%	>4000
Total		26	16		40 (20 to >4000)

^a^ Six swabs had multiple isolates within the same or different Gram morphology categories.

^b^ All isolates came from veterinarians from the surgery department.

### Questionnaires

Surveys were completed by 21 participants: 12 by veterinarians, eight by nurses and one participant preferred not to disclose their status. Out of 21 participants, the majority were 26‒50 years old (*n* = 17; 80%), two (9.5%) were 18‒25 years old and two (9.5%) were more than 50 years old. Twelve were female and nine were male. Use of their mobile phone in clinical areas was reported by 16 (76.2%) participants, 13 (61.9%) considered it an important tool and nine (42.9%) considered it essential. Five respondents (23.8%) admitted recently using their mobile phone in the bathroom and seven of 21 (33.3%) had never thought of mobile phones as being a potential fomite for HAIs.

Out of 21 participants, frequency of mobile phone cleaning was reported as at least once daily by three (14.3%), weekly by six (28.6%), monthly by six (28.6%), yearly by three (14.3%) and never by three (14.3%). Methods for cleaning mobile phones included an antiseptic wipe in 13 (61.2%), an alcohol swab in three (14.3%), a spectacle cleaning cloth in two (9.52%), the hem of a t‐shirt/scrub top in two (9.52%) and tissue or toilet roll in one (4.76%). Of 15 participants, reasons for not cleaning daily were reported as not thinking about it in eight (53.3%), concern about damage to the mobile phone in five (33.3%), considering good hand hygiene adequate in four (26.7%), lack of available learning resources in three (20%) and it taking too much time in one (6.7%).

**FIGURE 1:**
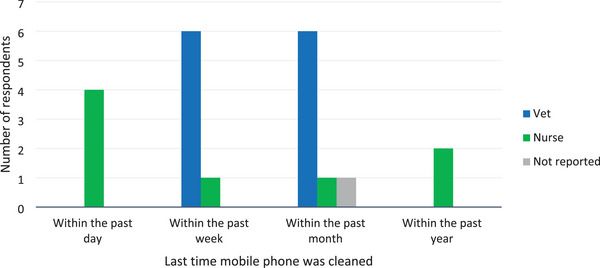
Bar chart showing the duration since mobile phones were last cleaned for both veterinarians and nurses whose mobile phones were swabbed for this study. Of note, no veterinarians had cleaned their phones within the previous 24 h.

**FIGURE 2:**
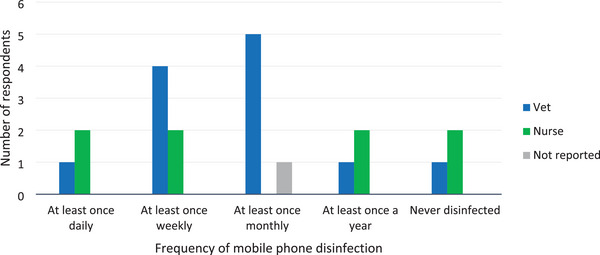
Bar chart showing the frequency of mobile phone disinfection for both veterinarians and nurses whose mobile phones were swabbed for this study. Although one veterinarian claimed they cleaned their phone at least once daily, this is at odds with the results shown in Figure 1, with no veterinarians having cleaned their phones within the 24 h prior to sampling.

Comparisons between veterinarians and nurses in the duration since their mobile phone was last cleaned and the frequency of routine mobile phone disinfection are shown in Figures 1 and 2. There was no significant difference between veterinarians and nurses in the use of an antimicrobial product to clean their mobile phone (antiseptic wipe or alcohol swab; *p* > 0.99) or recent use of their mobile phone in the bathroom (*p* > 0.99).

## DISCUSSION

This study found that among staff working in the theatre, patient preparation and anaesthesia areas of a veterinary teaching hospital, veterinarians were significantly more likely to have bacterial contamination of their mobile phones compared to nurses (62.5% vs. 6.7%; *p* = 0.0007). One reason for this could be that more nurses had cleaned their mobile phones within the past day compared to veterinarians, although the reported frequency of routine disinfection and selection of antimicrobial products to clean their mobile phones were similar between the two groups. This contrasts with the findings in human literature, where no significant differences were found between the incidence of contamination of doctors’ and nurses’ mobile phones.^9^, ^18^

Additionally, veterinarians from the surgery department had the highest incidence of mobile phone contamination (76.9%). Interestingly, similar findings have been reported in human healthcare, where surgeons’ devices had significantly higher levels of bacterial contamination than non‐surgical physicians.^13^, ^33^ Why this might be is currently open to speculation, but possible explanations include surgeons demonstrating poorer compliance with hygiene protocols and a greater number and variety of patient contacts.^13^, ^33^

The overall incidence of mobile phone contamination in this study was 41%. This is lower than the 68% previously reported for staphylococci alone in veterinary medicine^4^ and the 72.6%‒100% contamination rates reported in human healthcare.^3^, ^6^, ^7^, ^11^, ^17^, ^18^, ^20^, ^22^, ^23^, ^24^ This difference could potentially reflect a less sensitive sampling method or the death of bacteria between sampling and processing. Plain swabs moistened with saline were used in our study based on recommendations for environmental monitoring.^34^ A range of alternative approaches have been reported in other studies, including the use of a transport medium, direct inoculation of agar plates and electrostatic cloth which can be more sensitive.^3^, ^4^, ^9^, ^12^, ^19^, ^21^, ^34^ Two previous studies that used plan swabs reported close to 100% positive findings; however, these studies carried out DNA extraction for microbial identification, meaning we cannot compare our results.^17^, ^20^ Although our samples were processed within 4 h of collection, we cannot exclude the possibility there may have been some drying of swabs in transit to the laboratory, lowering the sensitivity of detection. While no prior warning was given of the study to prevent a Hawthorne effect, some participants may have cleaned their phones at the start of the work day, and these phones would be unlikely to culture positive when swabbed the same morning. Given the similar reported frequency of mobile phone cleaning in our study population to that reported in previous studies,^3^, ^6^, ^15^, ^20^ the authors believe the lower contamination rate reported here was unlikely due to better infection control practices alone but it may have been a contributing factor.

There was no significant difference between the incidence or amount of contamination between work phones and personal phones, despite work phones having more buttons and less streamlined cases. We believe this most likely reflects their reduced usage for voice calls only, as they have no touchscreen or internet connectivity. There may also be increased awareness and cleaning given their ‘work’ designation and a lack of concern regarding potential damage compared to personal phones.

Gram‐positive cocci were the most common contaminant bacteria, similar to reports in human healthcare.^3^, ^6^, ^9^, ^11^, ^13^, ^17^, ^19^, ^20^, ^21^ Although bacterial identification beyond Gram morphology was not performed in this study, typical examples referenced in previous literature include *Staphylococcus* spp., *Streptococcus* spp. and *Enterococcus* spp.^35^, ^36^ It remains unclear, however, what proportion are non‐significant commensals picked up from the phone user and what proportion might reflect potentially pathogenic microbes that could be important for HAIs, such as MRSA or MRSP.^3^, ^4^, ^11^, ^12^, ^14^, ^18^, ^20^, ^37^ A veterinary study, evaluating *Staphylococcus* spp. only, concluded that the majority of these Gram‐positive cocci were likely of human origin.^4^ Potentially greater concerns in our study were the Gram‐positive coccobacilli identified from two swabs (typical examples from previous literature include *Listeria* spp. and *Gardnerella* spp.), the Gram‐positive rods from two swabs (a typical example from previous literature includes *Corynebacter* spp.) and the Gram‐negative rods from two swabs (typical examples from previous literature include *Escherichia coli* spp., *Klebsiella* spp. and *Pseudomonas* spp.).^35^, ^36^ *E. coli* spp. and *Pseudomonas* spp. are bacteria of particular concern in veterinary MDR HAIs,^31^ meaning that the cultured Gram‐negative rods are more likely to reflect pathogens than commensals.^31^, ^35^, ^36^

Regular use of a mobile phone at work was reported by 76.2% of participants, 57.1% cleaned their mobile phone less than weekly with only 14.3% cleaning their mobile phones daily despite published recommendations on mobile phone hygiene and their effective decolonisation.^13^, ^16^ When mobile phones were cleaned, non‐antibacterial products were used by 23.8% of participants. Similarly high rates of mobile phone use and poor disinfection practices were found in a previous veterinary study^4^ and multiple human studies.^9^, ^10^, ^13^, ^23^ The major themes identified for poor mobile phone hygiene included not thinking about it; concern about damage from antiseptics or disinfectants; and considering good hand hygiene to be adequate. At the time of the study, mobile phone recommendations were not included in the hospital's infection control guidelines, which may have contributed to poor awareness and understanding. Good hand hygiene may reduce the risk of transmission from mobile phone to patient,^10^ but previous outbreaks of MDR HAIs linked to objects not directly contacting patients show that fomite control is still important.^28^, ^29^, ^30^ Therefore, good mobile phone hygiene is also necessary. The preliminary results of this study, showing significant contamination rates, were widely shared within the authors’ hospital to raise awareness. Hospital infection control guidelines were updated and confirmation was obtained from disinfectant wipe and mobile phone manufacturers of safe usage together to reassure staff.^38^, ^39^ This has led to a subjective improvement in hospital‐wide compliance with mobile phone hygiene measures. Similar awareness strategies have been proven to be effective in human hospitals.^14^

Infection control guidelines are well established and detail the importance of hand hygiene, environmental cleaning and fomite control.^40^, ^41^, ^42^, ^43^ However, the authors are aware of no guidelines that specify mobile phones and their risk as a fomite. One must consider that they are more ubiquitous and transported further than any other fomite, so have great potential to cause HAIs. The results of this study show that mobile phone hygiene recommendations should be included in hospital infection control guidelines. Previous recommendations that could be included are disinfecting mobile phones with 70% isopropyl alcohol wipes at least daily, before and after a patient interaction where the device is used, practising hand hygiene before and after using mobile phones, using on‐device reminders to prompt scheduled disinfection and for alcohol wipes to be widely available in clinical areas.^4^, ^8^, ^11^, ^14^, ^16^, ^44^

This study has several limitations related to its design, sample size and limitations imposed by the ethical review committee. The clinical significance and likely source of bacterial contamination could not be determined as speciation and antimicrobial sensitivity were not performed. Therefore, whether bacteria were commensal or pathogenic, human or animal origin could not be determined. Anaerobic bacteria may have been missed as no anaerobic cultures were undertaken. Non‐sterile gloves were used to hold mobile phones while swabbing and could have been a source of potential contamination; however, a fresh pair of gloves was donned for each phone to minimise this risk. The cross‐sectional design of the study provides a single snapshot of a cohort that may not represent the population of other hospitals. However, the sample cohort had a representative mixture of staff normally present in the theatre, patient preparation and anaesthesia areas, took place during busy working hours and sampled a majority of staff present at that time. Students’ mobile phones were not swabbed as the much larger sample size needed to obtain a representative student cohort was not possible with the available funding. Mobile phones were swabbed without prior notice to achieve as accurate a picture as possible of normal mobile phone contamination rates. Participation was not compulsory and some people meeting the inclusion criteria may have avoided sampling. The limited sample size overall and small numbers within some subgroups resulted in a limited ability to statistically compare some subgroups and may have led to type II error in the statistical analyses performed.^45^ Finally, individual mobile phone usage and cleaning habits could not be linked to specific bacteriology reports as ethical approval conditioned that questionnaires must remain fully anonymous and not be linked to individual phones. This prevented comparison of mobile phone habits between individuals with contaminated and uncontaminated mobile phones for potential contributing factors.

In conclusion, contamination of personal and work mobile phones was common among staff in this large veterinary teaching hospital, with veterinarians having a significantly greater incidence compared to nurses. Gram‐positive cocci were most prevalent, but Gram‐positive coccobacilli, Gram‐positive rods and Gram‐negative rods were also present. A minority of participants cleaned their mobile phones daily, some used potentially contaminated items such as t‐shirt and scrub suit hems to clean their phones, and some did not use an antibacterial product at all. To prevent mobile phones from acting as a fomite for HAIs, infection control guidelines could be updated to include mobile phone hygiene. Further research is warranted to determine the clinical significance of bacterial contamination of mobile phones and to evaluate their role in infection outbreaks.

## AUTHOR CONTRIBUTIONS

Kathryn Pratschke conceptualised the study and contributed to methodology, sample collection, data processing and original manuscript preparation. Owen Glenn contributed to methodology, sample collection, data processing and original manuscript preparation. Lisa Telford contributed to methodology and sample collection. All the authors reviewed and contributed to the final manuscript.

## CONFLICTS OF INTEREST

The authors declare they have no conflicts of interest.

## ETHICS STATEMENT

The study protocol and design were approved by the University of Edinburgh Human Ethical Review Committee (HERC 23‐008).

## Data Availability

There are no additional data available.
